# The dual roles of T cells and keratinocytes in seborrheic dermatitis: a narrative review

**DOI:** 10.1186/s40001-025-03005-4

**Published:** 2025-09-25

**Authors:** Yangfan Xu, Xiaoliang Tong

**Affiliations:** Department of Dermatology, The Third Xiangya Hospital, Central South University, Changsha, 410013 Hunan People’s Republic of China

**Keywords:** Seborrheic dermatitis, Keratinocyte, T cell, Etiology, Inflammation

## Abstract

Seborrheic dermatitis (SD) is a recurrent inflammatory skin disease primarily affects sebaceous gland-rich areas, particularly the scalp. Its characteristic clinical manifestations including dandruff, greasy crusting, and pruritus. These recurrent symptoms frequently impose significant psychological distress on patients. SD is classically associated with sebum secretion, Malassezia colonization, and individual susceptibility, whereas the critical roles of keratinocyte barrier dysfunction and T cell-mediated inflammation also constitute components in disease progression. In this review, we elucidate the mechanistic interplay between keratinocytes and T cells in SD development, proposing that their functional imbalance serves as a key driver of pathogenesis. We also systematically evaluate relevant therapies targeting keratinocytes and T cells, aiming to offer novel insights into SD pathophysiology and treatment.

## Introduction

Seborrheic dermatitis (SD) is a chronic recurrent inflammatory skin disease, which commonly affects seborrheic areas such as the scalp, face, chest and back [1, 2]. The primary clinical manifestation of SD include bright erythema covered with greasy scales or scabs, often accompanied by varying degrees of pruritus [3, 4]. SD can occur in individuals of all ages, including infants and the elderly [5, 6]. However, with the increase in high-fat and high-sugar diets [7], sleep disorders, and negative psychological factors [8], the global incidence of SD is increasing. This trend is accompanied by a shift toward younger-onset cases, with an estimated pooled global prevalence of 4.38% (95% CI: 3.58–5.17%) [9]. Clinically, SD often leads to persistent scalp pruritus, prolonged treatment courses, and frequent relapses [10, 11, 12], all of which contribute to substantial psychological stress in patients. This distress may, in turn, exacerbate disease progression [13, 14], creating a vicious cycle that complicates treatment.

Although the pathogenesis of SD is multifactorial, involving genetic predisposition, dysregulated lipid metabolism, and Malassezia overgrowth, current research consensus indicates that Malassezia colonization and its secretory products play a predominant role in SD development [2, 15, 16]. Malassezia is the normal microflora that colonizes the surface of skin, which hydrolyze sebum triglycerides into pro-inflammatory free fatty acids, thereby triggering cutaneous inflammation and barrier dysfunction [16]. Moreover, clinical evidence demonstrates that antifungal drugs significantly reduce scalp symptoms in patients with SD [17]. Although Malassezia exists as a commensal fungus in humans [18], its presence alone does not fully explain SD susceptibility, suggesting that additional factors likely contribute to disease development. These include compromised skin barrier function, immune dysregulation [19], hormonal influences [20], and environmental triggers such as stress, cold climates, and dietary habits [7, 21].

However, Zani demonstrated that no reduction in fungal counts was observed in patients with scalp SD treated with ketoconazole. In fact, an increase in fungal counts was detected in some patient samples. Despite this, the patients'symptoms improved [22], which suggests that ketoconazole may exert efficacy beyond its antifungal properties in the treatment of SD. Furthermore, they indicate that the pathogenesis of SD involves more complex mechanisms than previously understood.

The easily overlooked aspects of the etiology of SD are the barrier of the stratum corneum (SC) and immune activity. Keratinocytes, which compose the SC, are critical to the skin's physical barrier [23]. However, the broken barrier can lead to the invasion of Malassezia and trigger inflammatory reactions [24], which may act as both the “initiator” and “amplifier” of chronic inflammation in SD. The SC has been shown to exhibit hyperkeratosis in SD patients, and parakeratosis of keratinocytes has been identified as a key feature of dandruff production [25]. As well, T cells, as critical cells for immunoregulation, contribute significantly to the body's immune barrier. Elevated expression of T cell-mediated immune responses and inflammation-associated cytokines in patients further highlights the critical role of inflammation in SD pathogenesis [26].

We conducted the narrative review by searching PubMed for studies on SD and its relative researches, focusing on publications from the past 25 years. In this review, we will focus on the etiology of SD, emphasizing the dual roles of keratinocytes and T cells in SD. We will also emphasize the characteristics and interactions between these two cell types in the pathogenesis of SD, as well as therapeutic approaches targeting the SC and inflammatory response. By understanding the key roles of keratinocytes and T cells, we aim to provide effective strategies and rationale for the future treatment of SD.

## Proliferation, differentiation of T cells in SD

### Immunomodulatory of T cells in healthy skin

T cells play a pivotal role in maintaining immune barrier integrity, orchestrating protective immune responses, and regulating immune homeostasis [27]. As key mediators of adaptive immunity, T cells are mainly classified into CD4+ helper T cells (Th), CD8+ cytotoxic T cells and regulatory T cells [28]. Th17 cells enhance epithelial barrier function via secretion of IL-17A, IL-22, and IL-26, which stimulate antimicrobial peptide production [29–31] and keratinocyte proliferation [32], thereby reinforcing both physical and chemical defenses against bacterial and fungal invasion. In healthy skin, the IL-17 pathway is the key signaling that protects against pathogens and prevents overgrowth of Malassezia [33]. Notably, impaired IL-17 signaling leads to reduced antimicrobial peptide expression and exacerbates Malassezia colonization [33], which directly indicates T cells'dual role in immunomodulation and barrier protection.

Furthermore, Th cells can differentiate into different subpopulations, with non-pathogenic Th17 cells (expressing IL-10) clearing pathogens and pathogenic Th17 cells (expressing IFN-γ) leading to the development of inflammatory responses and tissue damage [33, 34]. Gringhuis demonstrated that dectin-1 in dendritic cells promotes the polarization of non-pathogenic Th17 cells upon recognition of the fungus [34]. This mechanism helps maintain immune homeostasis and prevents excessive inflammation and autoimmune damage during fungal infections.

### Involvement of T cells in the inflammatory response with SD

The inflammatory reaction is an important feature of SD [35], and similar to other inflammatory skin diseases characterized by scale and barrier damage, the epidermis of SD patients is infiltrated with T cells [36]. Susan et al. examined scalp biopsy specimens from subjects by Quantitative immunohistomorphometry and found that the number of epidermal CD4+ T cells and CD8+ T cells in the dandruff group were significantly higher than in healthy individuals [37]. This suggests that T cells proliferate and differentiate abundantly in the skin lesions of SD patients and are involved in triggering the inflammatory response associated with SD. The study by Kathy et al. examined not only biomarkers of differentiation and barrier integrity, but also inflammation-related markers, including interleukin-1α (IL-1α), IL-1 receptor antagonist (IL-1RA), and IL-8. The IL-1RA:IL-1α ratio was fivefold higher in the dandruff group than in the non-dandruff group, and the IL-8 amount was also fourfold higher in the dandruff group [38]. Elevated levels of both IL-1RA:IL-1α ratio and IL-8 underscore the significance of the inflammatory response in SD, and help illustrate the effect of T cells [39–41]. Mills et al. also investigated immune response-related genes and found that genes involved in the immune responses, defense against pathogens, and those encoding IL-1RA and IL-8 were significantly upregulated within dandruff lesions [42]. These findings were consistent with the clinical manifestations of inflammation in the scalp.

Mpzl3 (Myelin Protein Zero-Like 3) is a nuclear-encoded, mitochondria-localized immunoglobulin-like V-protein [43] that acts as a negative regulator of sebaceous gland size and sebocyte proliferation [44]. Mpzl3 knockout mice were shown to exhibit SD-like symptoms [45], with studies showing a twofold increase in the number of CD45+ leukocytes in the lesional skin compared to controls. Additionally, a significant increase in CD3+ T lymphocytes within the leukocyte-infiltrated areas was observed, along with a significant increase in the total number of γδ T cells and the γδ:αβ T cell ratio, although the total number of αβ T cells remained unchanged [26]. Similarly, they detected a significant increase as Skint1 expression by qPCR and an increase in IFN-γ, IL-17 expressed by γδ T cells. Since Skint1 is a key regulator of γδ T cell proliferation [46], its increase may explain the upregulation of γδ T cells. This study is also the first to propose the role of γδ T cells and IL-17 in the pathogenesis of SD, highlighting the integral involvement of T cells in the inflammatory response and disease development.

### Functional imbalance of T cells in SD

As previously mentioned, the Th17-mediated IL-17 pathway plays a critical role in preventing fungal overproliferation. However, IL-17AF deficiency attenuated Malassezia-induced epidermal thickening, indicating that it alleviates the inflammatory response rather than exacerbating it [33]. Additionally, they found that lack of T cells, especially γδ T cells, significantly reduced Malassezia-induced skin inflammation, this further suggests the central role for the IL-17 pathway and γδ T cells in stimulating the inflammatory response and promoting skin thickening aggravated by Malassezia.

Indeed, T cells may play a dual regulatory role in inflammation of SD, protecting the barrier from pathogens while also amplifying the inflammatory response and exacerbating symptoms. When this balance is disrupted, it leads to the induction of SD symptoms.

### SD in patients with HIV

SD is more prevalent in immunodeficient or immunosuppressed patients, such as those with AIDS, organ transplant recipients, and lymphoma patients. While the prevalence of SD is approximately 1–3% in general population, it can reach between 30 and 83% in HIV-infected individuals [47, 48]. AIDS, an acquired immunodeficiency syndrome, is caused by the virus that attacks T cells, leading to immune system dysfunction [49]. Studies have shown that SD usually occurs in the early stages of HIV infection, and as the disease progresses, the CD4+ T cell count decreases, and SD symptoms worsen. This suggests that SD can be considered as a marker of the progression of the AIDS [50]. All these results were associated with T cells, and the number of T cells was strongly correlated with the symptoms of SD in AIDS [50–52], also confirming that the proliferation and differentiation of T cells are closely implicated in SD.

## Proliferation, differentiation of keratinocytes in SD

### The structure and function of the stratum corneum

The stratum corneum (SC), the outermost layer of the epidermis, primarily derives its critical barrier function from terminally differentiated keratinocytes [53]. The SC is dense and flexible, and has a complete lipid membrane, rich in keratin and filaggrin [54], which can resist external environmental stimuli, such as ultraviolet light, oxidative stress and friction [55]. Meanwhile, it prevents the loss of water and nutrients through its semi-permeable membrane function, known as epidermal permeability barrier (EPB) [56]. The EPB is mainly composed of a bilayer of lipid molecules, including ceramides, cholesterol, fatty acids and triglycerides, which block the intercellular space of the SC [57, 58]. These lipids are indispensable and play a crucial role in maintaining the barrier function. Any changes in the gene expression of key lipid biosynthesis and metabolic pathway involved in EPB composition will lead to the destruction of barrier function.

Tight junction proteins between corneocytes regulate paracellular permeability [59], while antimicrobial peptides (β-defensins, cathelicidin) and cytokine-mediated immune surveillance embedded in this layer provide innate defense against pathogens [60]. This multifunctional keratinocyte-mediated barrier system dynamically balances epidermal homeostasis through continuous desquamation and regeneration processes regulated by pH, proteases, and lipid metabolism [61].

Pathological changes in the skin of SD patients: in the scalp SC of healthy individuals, keratinocytes are compactly arranged and exhibit normal morphological features, with standard desmosomal structures. However, in the ultrastructure of scalp SC from SD patients, keratinocytes were less tightly arranged due to thick intercellular lipids in the intermediate layer. The number of desmosomes was significantly reduced, and some samples lack desmosomes entirely. Additionally, Malassezia infiltration is present, and keratinocytes exhibited intracellular curling in some sample, whereas some samples had a high degree of interlaced membrane structure, which showed a kind of"scared"structure [62]. However, the most prominent pathological structure of keratinocytes is parakeratosis and the presence of large numbers of intracellular lipid droplets [38]. Parakeratosis reflects excessive keratinocyte proliferation in the SC of SD patients, and one of the characteristic manifestations of dandruff is the presence of lipid droplets in the keratinocytes, the excessive accumulation of lipid droplets in the cells is a typical consequence of pathologic keratinization [62, 63]. Overproliferation of keratinocytes is a major contributor to dandruff symptoms in patients.

The lipids enriched in keratinocytes further lead to the destruction of the SC. Studies showed that unsaturated fatty acids increased calcium influx into keratinocytes, delayed the recovery of skin barrier function, and induced epidermal hyperplasia [64, 65]. Notably, the fungal lipase LIP1 from *Malassezia globosa* has been shown to exacerbate this process. LIP1 hydrolyzes sebum triglycerides to release free unsaturated fatty acids, which penetrate the SC and disrupt its lipid bilayer organization [66].

### Impaired barrier protection of keratinocytes in SD

Kathy et al. examined biomarkers of differentiation and barrier integrity by scalp biopsy specimens from patients with dandruff [38]. Differentiation markers included keratins 1, 10, and 11 (K1, K10, and K11) and involucrin, while barrier integrity was measured by measuring SC lipids and human serum albumin (HSA) [67]. Immunoreactivity to K1, K10, and K11 antibodies was sixfold lower in SC from dandruff patients than in non-dandruff controls, though involucrin reactivity showed no significant difference [38]. This suggests a substantial reduction in keratin content in SC from dandruff patients, which is also consistent with the parakeratosis due to hyperproliferation of keratinocytes, as mentioned earlier. They also found a onefold increase in HSA levels and significant reductions in total ceramide and free fatty acid content compared to controls, while cholesterol content remained unchanged. Notably, the study also reported decreased levels of sphingolipid precursors and total sphingolipid bases in dandruff SCs [38].

Mills et al. collected scalp biopsies from dandruff patients and normal controls for bioinformatics analysis. Their results demonstrated significantly reduced expression of genes involved in cholesterol, triglyceride, and fatty acid synthesis in dandruff patients. Notably, the gene encoding fatty acid synthase, which is the rate-limiting enzyme for fatty acid synthesis, was decreased by approximately 50% in the dandruff group [42]. This downregulation presumably contributes to the impaired integrity of the EPB barrier in SC. Furthermore, the study revealed excessive upregulation of genes associated with both cell proliferation and epidermal differentiation in dandruff patients, further suggesting that epidermal barrier integrity and homeostasis are indeed compromised.

The dynamic balance of the composition in the SC is essential for maintaining skin barrier function. However, the hyperproliferation of keratinocytes in SD suppresses terminal differentiation markers [68]. This leads to significantly reduced expression of barrier proteins, antimicrobial peptides, and β-defensins [69, 70], which ultimately impairs skin barrier and antimicrobial function.

### Metabolic interactions between keratinocytes and the microbiome

Toll-like receptors (TLRs) in keratinocytes serve as critical sentinels of cutaneous innate immunity, mediating pathogen recognition and orchestrating diverse immune responses through finely regulated signaling pathways [71]. In particular, keratinocytes express low levels of TLR2, but it has been shown that TLR2 can recognize receptors when cells are invaded by pathogens as a way of maintaining the homeostatic role of the skin [71, 72]. In addition, β-glucan released by the superficial fungus Trichophyton rubrum was also shown to significantly stimulate TLR2 expression by HaCaT cell [73], further suggesting a recognition relationship between keratinocytes and fungi. Since β-glucan is an important component of fungal cell walls [74], this may represent an important pathway for the recognition of Malassezia by keratinocytes in SD, potentially triggering subsequent inflammatory response could be explored.

Aryl hydrocarbon receptor (AhR) is an environmentally sensed exogenous receptor and ligand-activated transcription factor essential for skin immune homeostasis and barrier maintenance [75]. Emerging evidence indicates that symbiotic microorganisms of the skin can activate the AhR of keratinocytes, thereby modulating barrier function and facilitating pathogen defense [61]. In addition, the intrinsic AhR–Ovol1–Id1 regulatory axis in keratinocytes has been demonstrated to maintain epidermal and immune homeostasis during skin inflammation [76]. Activation of AhR significantly reduces the abnormal differentiation and proliferation of keratinocytes by IL-24 and improves the function of the skin barrier [77]. Notably, Malassezia yeast cultured in tryptophan-containing media produces multiple AhR ligands [78]. Clinical studies have identified significantly higher levels of three of AhR have been isolated from the skin of SD patients than healthy individuals [79, 80]. While these findings may represent Malassezia-derived AhR ligands may serve either as: (1) a microbial self-protective mechanism to mitigate inflammation and barrier disruption, or (2) pathogenic factors exacerbating epidermal differentiation defects and inflammation. However, we have no specific knowledge of the exact mechanism, and more direct evidence is needed to prove this.

Consequently, keratinocytes exhibit a dual role: while normally serving as protective cells against pathogens, they may paradoxically contribute to increased microbial invasion susceptibility. Perhaps more experiments are needed to support these points.

## Interaction between keratinocytes and T cells

### Keratinocytes activate and amplify inflammation

Keratinocytes and T cells, as important links in the pathogenesis of SD, do not exist in absolute isolation but interact with each other, contributing to the development of SD. Recent RNA-seq results show that Malassezia induces keratinocytes to secrete highly expressed IL-23 and promotes differentiation of pathogenic Th17 cells, which in turn exhibit a pro-inflammatory response and produce cytokines such as IL-17A, IL-17F, IL-22, and IL-23R [81]. Similarly, TLR2 in keratinocytes recognizes fungal cell wall components, leading to the secretion of inflammatory factors such as IL-1β, IL-6, and TNF-α by NF-κB and mitogen-activated protein kinase (MAPK) pathways to amplify inflammation [82]. The role of keratinocytes in amplifying inflammation in SD could be explored. Besides, the increase of free fatty acids in SC that occurs in SD also activates IL-1β and NF-kB, which promotes the inflammatory response [19] and creates a “lipid–inflammatory” vicious cycle.

MiR-146a, a microRNA extensively studied for its role in immune regulation and inflammatory responses, is involved in the regulation of immune responses in keratinocytes [83]. Meanwhile, miR-146a has been shown to be expressed at elevated levels in inflammatory skin diseases such as acne, psoriasis and atopic dermatitis (AD) [84–86]. By targeting key adaptors in the TLR and IL-1 receptor signaling pathways, such as interleukin-1 receptor-associated kinase 1 and TNF receptor-associated factor 6, miR-146a inhibits pro-inflammatory mediator production and blocks TLR signaling [83]. This represents that miR-146a may function as a negative feedback regulator to prevent excessive inflammation in keratinocytes. However, while many studies have documented the immunomodulatory role of miR-146a in chronic inflammatory skin diseases, its role in SD remains unexplored. Whether diminished miR-146a promotes immune dysregulation in SD by enabling keratinocytes to amplify T cell-mediated inflammation remains an open question that demands further exploration.

### T cell-mediated regulation of keratinocyte function

Besides, T cells can also influence the differentiation and proliferation of keratinocytes. In chronic diseases, T cells stimulate keratinocyte proliferation through the production of TNF-α and IL-17, and IFN-γ and IL-22 [87]. IFN-γ produced by Th1 cells supports the recruitment of more Th1 cells and amplifies the stimulation of keratinocytes, IL-17 promotes neutrophil migration into the skin and stimulates the production of antimicrobial peptides by keratinocytes [88, 89].

**Fig. 1:**
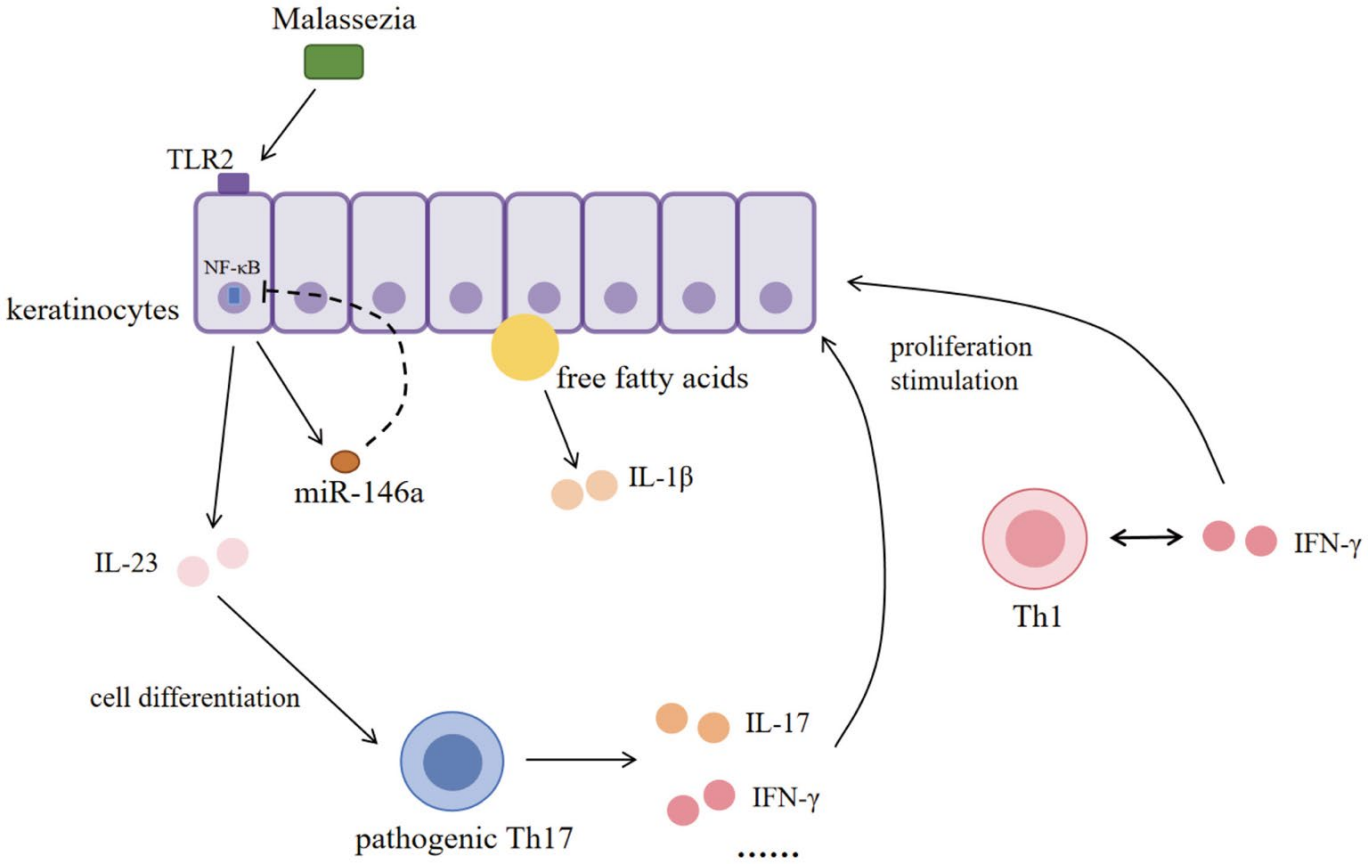
Schematic illustration of interactions between Malassezia, keratinocytes, and T cells. The dotted curve represents the inhibitory effect

In summary, hyperproliferation and parakeratosis of keratinocytes combined with reduction of lipid content lead to barrier dysfunction. This dysfunction can lead to inappropriately accelerated proliferation of the epidermis and an increase in inflammatory markers, further contributing to the parakeratosis of keratinocytes in the SC and triggering an autocatalytic cascade of inflammation that disrupts normal cell proliferation and differentiation (Fig. 1) [90].

## Treatment

### Treatments for reducing dandruff

In patients with SD, hyperproliferation of keratinocytes causes keratinocyte accumulation and parakeratosis, which leads to dandruff production, and Malassezia strongly induces this process. Therefore, treatments for dandruff are typically categorized as antifungal drugs and keratolytic agents (Table 1).

**Table 1:** Comparison of treatments for scale and inflammation in SD

Treatment	Mechanism	Advantage	Common adverse effects
Dandruff			
Antifungal drugs	Targeting fungal cell membranes to disrupt fungal integrity	Dual antifungal and anti-inflammatory action, improvement of microecological environment	Burning, irritation, photosensitivity, systemic absorption risk
Keratolytic agents	Promoting exfoliation of the over-keratinized stratum corneum and regulating sebum secretion	Increased penetration of complementary therapies	Burning, irritation, dryness, contact dermatitis, erythema
Inflammatory			
Corticosteroids	Anti-inflammatory, immunosuppressive, and vasoconstrictive properties	Rapid symptom control, versatility in formulations	Burning, atrophy, telangiectasia, steroid-induced rosacea, infection, hypopigmentation
Calcineurin inhibitors	Suppressing T cell-mediated inflammation	Dual anti-inflammatory and barrier-preserving effects	Burning, stinging, erythema, upper respiratory infection
Roflumilast	Reducing pro-inflammatory mediators by inhibiting cyclic adenosine monophosphate degradation	Highly effective phosphodiesterase 4 inhibitor	More trials are needed to determine its durability and safety

### Antifungal drugs

Antifungal drugs are a cornerstone in the management of SD. These drugs, including azoles, ciclopirox, selenium sulfide, and piroctone olamine, primarily target fungal cell membranes by inhibiting ergosterol synthesis (azoles) or disrupting membrane integrity (ciclopirox) [91, 92]. By reducing Malassezia proliferation and associated lipase activity, which releases pro-inflammatory free fatty acids, antifungals mitigate inflammation, scaling, and erythema.

The treatment of antifungal drugs is divided into internal and external use. Topical antifungal therapy is the main treatment for SD, one of the most commonly used drugs is ketoconazole. Vinicius concluded that ketoconazole has immunosuppressive activity in addition to antifungal activity, as well as the ability to affect lipid metabolism in Malassezia by affecting stratum corneum lipid distribution [93], leading to symptomatic improvement. Moreover, ketoconazole is able to achieve the same clinical effects as hormonal drugs, but with fewer side effects [94]. Thus, antifungal drugs have broad application prospects in SD treatment.

Antifungals drugs offer dual antifungal and anti-inflammatory effects, addressing both the microbial trigger and downstream inflammation. Topical formulations can avoid corticosteroid-related adverse effects such as atrophy or telangiectasia [95]. For example, ketoconazole shows efficacy comparable to low-potency steroids in improving symptoms [96]. However, antifungal monotherapy may be insufficient in severe or recalcitrant cases, necessitating adjunct therapies [97]. Furthermore, systemic absorption risks warrant caution, though topical agents generally exhibit favorable safety profiles [98].

### Keratolytic agents

Keratolytic agents, including salicylic acid, sulfur, urea, and zinc pyrithione, primarily function by promoting the desquamation of hyperkeratotic SC, reducing scale accumulation, and modulating sebum secretion. Salicylic acid, a β-hydroxy acid, dissolves intercellular lipids and corneocyte adhesions, facilitating the removal of dead skin cells [99]. As a common moisturizer and keratolytic agent, urea also has antifungal properties, which can effectively enhance the penetration of topical drugs while reducing the symptoms in SD patients [100]. This lays the foundation for the combination of urea and other drugs in the treatment of SD.

Keratolytic agents are suitable for long-term use and also avoid the side effects of long-term corticosteroid application. Meanwhile, they can enhance the penetration of complementary therapies by removing scales and debris, thereby improving the overall therapeutic effect [100]. These agents show a synergistic effect in reducing flaking and itching, not only exfoliating the skin, but also inhibiting the proliferation of Malassezia, addressing both hyperkeratosis and microbial overgrowth.

### Treatment to reduce the inflammatory response

Inflammatory reactions are an important cause of symptoms in patients with SD, and conventional anti-inflammatory treatments usually include corticosteroids and calcineurin inhibitors, along with the newer agent roflumilast (Table 1).

### Corticosteroids

Corticosteroids are effective due to their anti-inflammatory, immunosuppressive, and vasoconstrictive properties [101]. Mechanistically, they bind to intracellular glucocorticoid receptors, modulating gene transcription to suppress pro-inflammatory mediators such as cytokines, chemokines, and adhesion molecules [102]. They also inhibit phospholipase A2, reducing the synthesis of prostaglandins and leukotrienes, and stabilize lysosomal membranes to limit the release of inflammatory enzymes [103].

Corticosteroids provide rapid relief from inflammation, pruritus, and erythema, making them particularly effective during acute exacerbations [104]. Topical corticosteroids (TCS) are often co-administered with antifungal drugs in SD treatment because the combination better controls the progression of the disease. However, chronic application, especially of high-potency corticosteroids, risks cutaneous atrophy, telangiectasia, and steroid-induced rosacea [105–107]. These effects result from collagen degradation and dermal thinning caused by suppressed fibroblast activity [108]. Additionally, abrupt discontinuation may trigger rebound inflammation or even hyperalgesia [109], necessitating gradual tapering. Moreover, immunosuppressive effects may increase susceptibility to bacterial or fungal infections, emphasizing the need for cautious use in infected or intertriginous areas [110].

### Calcineurin inhibitors

Calcineurin inhibitors (CNIs), including tacrolimus and pimecrolimus, target the immune dysregulation involved in the pathogenesis of SD. CNIs bind to intracellular immunophilin receptor, forming a complex that inhibits calmodulin phosphatase activity, thereby suppressing T cell activation and inflammatory cytokine production [111]. By attenuating T cell-mediated inflammation, CNIs alleviate erythema, scale, and pruritus without compromising the skin barrier, which is often disrupted in SD.

In contrast to TCS, CNIs do not induce cutaneous atrophy or rebound flares, making them suitable for long-term or intermittent use on sensitive areas like the face [112]. Studies have shown that 0.1% tacrolimus ointment exhibits comparable efficacy to 1% hydrocortisone ointment for SD treatment, while requiring fewer applications due to more rapid symptom resolution [113]. Furthermore, CNIs result in longer-lasting symptom relief and lower relapse rates compared to traditional therapies [114]. Transient burning, stinging, or erythema at the application site is common, particularly during initial use, potentially affecting patient adherence [115]. However, while prolonged use of systemic CNIs is associated with risks such as skin malignancy and infection, topical formulations are generally considered safer [116].

### Roflumilast

The new research finding that 0.3% roflumilast topical foam is a highly effective treatment of seborrheic dermatitis (approved by the FDA on December 15, 2023) [117]. Roflumilast inhibits the proliferation of CD4+ T cells and reduces the number of Th1, Th2 cells, thereby reducing the production of IL-4, IL-5, IL-13, IPN-r, lymphotoxin and the levels of allergen induced eosinophils [118]. However, longer trials are needed to determine the durability and safety of roflumilast in SD.

## Conclusions

Current research on SD pathogenesis primarily focuses on Malassezia infection, while individual susceptibility factors remain relatively understudied. The damage of EPB may represent a cause of this susceptibility, and both endogenous and exogenous factors can disrupt EPB homeostasis, resulting in the damage of the epidermal SC structure. Barrier dysfunction facilitates the invasion of Malassezia and its metabolites, while SC promote inflammatory responses that exacerbate SD symptoms. Therefore, these findings highlight the crucial roles of keratinocytes and T cells in SD pathogenesis.

Similarly, we hypothesize that the pathogenesis of SD results from an imbalance in the function of keratinocytes and T cells, both of which exhibit dual protective and pathogenic aspects in SD. Only when this homeostatic balance is disturbed does commensal Malassezia become pathogenic, synergistically amplifying disease progression factors.

The significant down-regulation of genes related to lipid metabolism, including those involved in fatty acids and cholesterol, was observed in SD patients. Additionally, the levels of ceramides and free fatty acids were significantly reduced in scalp samples from SD patients. All these findings could provide strong evidence for EPB damage, while the high expression of genes encoding inflammation-related cytokines and the increased content of cytokines and T cells in the samples also indicated the important role of inflammatory response in SD. However, there is not always a complete alignment between gene expression and protein phenotypic expression. For instance, although cholesterol-related genes were significantly down-regulated, no significant changes were observed in SC, which may be attributed to gene transcription and its modification, as well as the complex feedback regulation mechanism in the organism [42]. Perhaps more studies are needed to prove this relationship. Nevertheless, the genes in lipid metabolism along with cell proliferation and differentiation are consistent with the phenotype, so they can be considered as reliable conclusions.

As we mentioned earlier, current treatments for SD are limited and associated with high recurrence rates, thus novel and effective therapies are necessary. It may be possible to consider the topical application of specific inhibitors of AhR and TLR2 to block the cascade amplification between keratinocytes and T cells, and thus control the disease progression. Furthermore, as previously mentioned, some mechanisms have not yet been explored in the context of SD (e.g., miR-146a). Further deeper studies are necessary to determine whether these mechanisms contribute to SD pathogenesis and to inform future therapeutic strategies.

## Limitation

First, while we have synthesized key findings from SD-related literature, our search strategy may not have captured all relevant studies on SD. Second, although we incorporated selected research on AD and psoriasis due to their shared pathological features with SD, part of these findings require direct validation in SD as their applicability to SD pathogenesis remains uncertain. AD and psoriasis ultimately need to be distinguished from SD. These limitations constrain the generalizability of our conclusions and highlight the need for more SD-specific investigations. We hope that this review will provide valuable insights into SD pathogenesis and aids in the development of innovative treatments.

## Data Availability

No datasets were generated or analysed during the current study.
